# Hygiene of the Turkish Army

**Published:** 1857-10

**Authors:** J. N. Radcliffe

**Affiliations:** late of the Staff of His Highness Omar Pasha.


### ORIGINAL COMMUNICATIONS.

### THE HYGIENE OF THE TURKISH ARMY.

By J. N. RADCLIFFE, M.R.C.S.Eng., late of the Staff of His Highness Omar Pasha.

(*Concluded from* p. 157.)

### IV.—THE CAMP.

A TURKISH camp has commonly a very pleasing aspect, the Turkish soldiers being well versed in those arts which give order and neatness to an encampment. The tents are pitched well and with great regularity; they are not crowded together, nor fixed too far apart, and generally the ground in front and in the intervals of the lines of tents is carefully cleaned and smoothed; or if it be apt to retain much moisture, it is neatly paved with stones, forming a capital stand for the arms, and enabling the men, in wet weather, to enter their tents without dragging along with them heavy clods of tenacious mud. The latrines are placed sufficiently in the rear, and they are usually made with great care. During the Crimean war no other section of the allied forces equalled the Turkish troops in the construction of latrines. A deep hole is dug, and this is in great measure covered in, a small aperture only being left, above which is pitched a latrine tent, or, if brushwood be at hand, a hut is often erected. Thus the eye is not offended by those huge and filthy holes which were in common use among the soldiers of the English, French, and Sardinian armies, and the stench from the latrines is materially diminished.

But order and neatness are not equivalent to a good hygienic condition of a camp; and while, on the one hand, a well pitched Turkish encampment might be held up as a model to other armies, on the other hand the Turkish soldier has certain pestilent habits which render an encampment as offensive as can well be imagined. He scatters offal about most carelessly, and he has a singular aversion to the use of latrines, notwithstanding his care in their erection, and if practicable, he will almost invariably retire to the verge of the encampment; hence it happens that its immediate vicinity, as the nostrils and the eyes too readily detect, is commonly defiled in the most disgusting manner.

In the fine seasons of the year, and when near wood, the Turkish soldier will, with great ingenuity, embellish and add to the comforts of an encampment by erecting bowers and shades of foliage, beneath which he delights to recline during the warmer hours of the day, in preference to remaining within the hot tent. A Turkish encampment, under such circumstances, is a singularly attractive spectacle.

After the advance upon the Tchernaya in May 1855, the Turkish forces took up a position on the low ridge of hills which traverse the plain of Balaklava. The white tents were pitched with great regularity upon the eastern face of the ridge; and upon the summits of the hills, and in front of the different groups of tents, were erected bowers and shades of the most varied character, and formed of branches of shrubs and trees obtained from the wooded hills on the right. There were large sheds, open on every side, and elaborately constructed bowers, and a multiplicity of small huts. Even the cooking places were hedged in with foliage, or hutted over. The aspect of these constructions of rich green foliage was very beautiful; and even when the fresh colour began to fail, and it became spotted with brown and yellow as the leaves faded, the bowers and huts had still a most pleasing effect upon the eye.

The Turkish soldier, when on the march, shows also considerable ingenuity in erecting temporary shelters of boughs and foliage to protect himself from the heat of the sun by day, and from the wind or dew by night.

The tent used in the Turkish army is the ordinary bell-tent. It is formed of two layers of strong cotton; and, when properly pitched, it has within, at the base, a diameter of about fourteen feet. It is well made, and upon the whole it resists wet well.

The regulation number of men to each tent is ten, but the actual number is almost always in excess, and not unfrequently it is fifteen.

The Turkish soldier makes himself very comfortable within a tent. If he be encamped for any length of time upon one spot, or even if it be for a short period only, he will contrive to arrange his tent so that he may have in it the height of compaigning comfort. He displays excellent skill in the formation of a floor that presents few asperities to his feet as he walks upon it shoeless, or to his sides when he is laid down; he banks up the flaps well with earth, and trenches the tent round, so as to diminish the chances of wet or of wind intruding; and in rainy or cold weather, he will ingeniously build, of loose stones and mud, a small fireplace within his tent, so that he may conduct there the whole process of his cooking. He possesses also, in a high degree, the art of making his tent as little permeable to the weather as possible, by walling it round, or sinking it in the earth, during winter.

The Turkish soldier has, however, very imperfect notions of the benefits of pure air. He likes to bask in the sun and lounge in the open air in fine weather; but he cares little about the condition of the atmosphere he breathes, and, it mattered not how foul the air in his tent might be, it was rare to see him take measures to purify it.

A bell-tent, although probably a convenient tent for carriage and for pitching, is not capable of thorough and effective ventilation; and this becomes a question of some moment, when a tent is pitched for a considerable length of time upon one spot. It is usual, and indeed necessary, to raise the flaps of a bell-tent to ventilate it, but, if the tent be well banked, this is an awkward and most troublesome method; moreover, it can only be had recourse to in fine weather, for in wet or windy weather it leads to too great exposure and discomfort.

The defective ventilation of tents, an evil which is dependent upon the necessity for making them as weather-proof as possible, and which is found, more or less, in tents of every form, is important, inasmuch as it intensifies the action of emanations arising from decomposing animal and vegetable matter, the most general and powerful source of atmospheric pollution to which a camp is liable. In the crowded and confined tent the soldier suffers most from an atmosphere thus vitiated, particularly when at night he is sleeping upon the ground.

The double-poled, or Arab tent, which was used by several of the French regiments in the Crimea, seemed to be, hygienically considered, a much better form of tent than the bell-tent. The former is larger and more commodious within, and its interior can, on either side, be thrown fully open to the air. It is doubtful, however, how far tents of this form admit of being so readily pitched and managed as the bell-tent, and whether they are so well calculated to stand rough weather.

Attempts have been made to obviate the defective ventilation of the bell-tent by apertures placed at the summit and variously guarded; but this method of ventilation has not been so successful as to lead to its general adoption, and it is liable to some objection in stormy weather.

During the Crimean war, none of the soldiers of the allied forces appeared to get on so comfortably under canvass as the Turkish soldiers. The English, French, and Sardinian soldiers put up with the mode of life as a necessary evil of campaigning, and they made themselves as comfortable as practicable under the circumstances; but the Turkish soldiers, although having their peculiar grievances, seemed to settle down more comfortably under canvass than the soldiers of the other forces, and to be actually more comfortable.

This probably arose from the domestic habits of the Turk being such that in a tent-life, and in camp-life generally, as it affects him, there is not that great change in the mode of living that there is among the English, French, and Sardinian soldiers.

It is customary among the class from which the soldiers and the bulk of the inferior officers of the Turkish army is derived, for a whole family, though large, to occupy during the day, and also at night, one room only of a house, even if the house contains several rooms, and in that room to live, cook, and sleep. Moreover, carpets or thin cushions, placed upon the floor, constitute the sole necessary and the usual articles of furniture, and serve for seats by day and beds by night. The cooking apparatus is equally simple, two or three metal stew-pans and a frying-pan serving for a family; and the articles of food differ in no respect from those issued to the army. Hence it happens that, as a soldier, the Turk finds little in the crowded tent that is at variance with his habits; he has in the carpet which is issued to him a full allowance of household furniture; in every mess there is an amount of cooking utensils almost equal to, and in no way differing from, what he has been accustomed to in his own dwelling; the food which he receives as rations is in character and quality such as he has always been used to, and he can cook it in those fashions to which he has been habituated, and which he prefers most.

That this comparatively slight degree of change between the habits and method of living in ordinary life and that of the soldier's life, has no small influence in contributing to the efficiency of the Turkish soldier in the field, there can be little doubt.

### V.—THE HOSPITAL.

My acquaintance with Turkish Military Hospitals was confined solely to the temporary hospitals in the Crimea. A description of the hospital at Balaklava will give a correct idea of the general character and management of the Turkish Field Hospitals during the Crimean war.

The hospital at Balaklava consisted of twenty-eight huts, which were situated in an almost central position within the lines on the heights east of the village. The position of the hospital was good, although not easy of access, for it was at an elevation of nearly six hundred feet above the lowlands, and it was freely open to the winds, without being too much exposed; there was every facility for drainage and cleanliness, and numerous and copious springs of water were in the vicinity.

The huts were of British construction; they were built of $\frac{3}{4}$ths of an inch planks, and the roofs had, with few exceptions, a water-proof felt covering. Twenty-four of the huts were of the same form and dimensions as those in use among the British troops, and they measured 28 feet in length, and 16 feet in breadth; the height of the side walls was 6 feet, and that of the summit of the roof was from 11 to 12 feet. Within was a central pathway, 3 feet in width, which ran from end to end, and on each side was a wood flooring raised 6 inches from the ground, and $6\frac{1}{2}$ feet in width. Each of the huts had two windows, one being placed at each extremity. The windows were 3 feet square, they were elevated six feet above the floor, and the sashes were glazed and moved on a transverse axis. There was a single door to each hut, and it opened in the majority towards the south—the huts being arranged so that the longitudinal axis was almost directly north and south.

The huts were placed very close together, the intervening spaces not measuring more than six feet, and each hut was trenched round, but in a very imperfect manner; moreover, the trenches had no communication with each other, and no proper outlets.

The huts were erected early in 1855, and during the hot season the wood warped and cracked considerably. The walls were neither painted nor white-washed within or without, and in the space beneath the flooring swarms of mice and rats found shelter.

There was no effective mode of ventilating huts of this construction without discomfort to those within, and constant care and watchfulness were necessary in order to keep the walls and roof in the condition requisite to exclude wind and rain. In all such attentions as were necessary for the preservation of the integrity of the huts, the hospital authorities and the Turkish soldiers were wanting; and the previous condition of the walls during the summer and autumn of 1855 was perhaps occasioned less by the slightness of their construction than by the carelessness of the Turks. The means for any trifling repair, even to the extent of a few nails, were rarely at hand; and the only method adopted to prevent the intrusion of wind and rain through the numerous crannies of the warped wood-work was by pasting paper over them.

The remaining huts were of small dimensions, and they were well constructed, and painted externally. These huts were appropriated to sick officers and the resident surgeons.

Each of the large huts accommodated fourteen patients, and to each hut were attached two orderlies, who lived and slept along with the sick of whom they had charge. No bedsteads were used, the patients being laid upon good flock beds of about four inches in thickness, which were placed (after the fashion of the East) upon the floor. The beds were covered with thick, white, and soft cotton, and each bed was provided with a sheet of the same material, and a heavy white coverlid. Additional covering might be had if required, but it was rarely necessary, as each patient had his great coat along with him. The beds and bed-linen, and indeed the patient's dress generally, were almost invariably kept clean, and the principal portion of the materials being white, the aspect of the occupied huts was usually neat and satisfactory. The bedding and clothing of the patients were much less infested by insects than might have been anticipated from the construction of the huts and the peculiar habits of the men.

At the head of every bed was suspended a ticket, upon which was noted the name, age, father's name, province, regiment, battalion, and time of admission of the patient.

The hospital dress consisted of a long, blue, wide-sleeved, loose coat, of indifferent texture, in addition to the usual under-clothing of thick cotton.

Each hut was provided with two large and very useful basins for the general use of the occupants, a sufficient number of small basins for food, cups, plates, spoons (all of which utensils were made of metal), sponges, a charcoal-burner, and a tub or a number of bottles for containing water.

Within the huts a considerable degree of cleanliness was maintained, but external to them cleanliness was little heeded. The vicinity of the hospital was most foully littered with filth, in consequence of the abominable habit which the Turkish soldiers have of avoiding latrines.

The latrine for the general use of the patients was placed about thirty feet from the huts. It was a capacious trench of about twenty feet in length, and a rough construction of planks had been erected above it. To the left of the latrine, at a distance of about thirty feet, was the cooking-house, and in its rear a hut for stores. In the space between these buildings and the latrine, it was customary for many months to kill the oxen and sheep required for the use of the hospital. The whole of the blood from the slain animals was allowed to run, and the refuse was thrown into a hole behind the store-hut. This hole was distant about fifty feet from the hospital huts, and it was full to overflowing of decomposing animal matter, from which arose a formidable stench.

Notwithstanding the contiguity of this sink of abomination and of the latrine, and that the ground was in a most filthy state, a short time previously to the removal of the Turkish forces from the neighbourhood of Balaklava to Mingrelia, several tents were pitched, for the accommodation of sick officers, in the open space between the cooking and store-huts and the latrines. Two detached latrines were also formed in the rear of these tents, and close to the walls of the store-hut.

The bed-pan used in the hospital was an iron vessel of very inconvenient form and size. This utensil was, however, too rarely had recourse to, and it was often painful to witness patients, seriously prostrated from disease, conducted to the latrine by an orderly.

The huts were heated when necessary by stoves, or by braziers, termed *mangals*, and fed with charcoal. The charcoal is ignited in the open air, where it is kept until it becomes incandescent, when it is in a state fit for use in an apartment.

The *mangal* is a very objectionable means of obtaining warmth, particularly in crowded quarters or tents, for the men, in order to secure the greatest degree of heat, invariably close every accessible aperture by which air can find admission; and under such circumstances, the fumes of burning charcoal, however carefully it may be managed, become an important additional source of atmospheric vitiation.

The food issued to the patients was generally good, and it was sufficient in quantity. The daily diet-scale of the hospital was as follows:—

*Full Diet.* Bread, 200 drachms ($22\frac{1}{2}$ oz.); rice, 92 drachms ($10\frac{1}{3}$ oz.); "yagh," 8 drachms (nearly 1 oz.); meat, 100 drachms ($11\frac{1}{4}$ oz.); onions and other vegetables in variable quantities, and salt.

*Half Diet.* Bread, 100 drachms ($11\frac{1}{4}$ oz.); rice, 80 drachms ($9\frac{1}{2}$ oz.); "yagh," 8 drachms (nearly 1 oz.); and meat, 50 drachms ($5\frac{1}{2}$ oz.)

*Low Diet.* Bread, $33\frac{1}{2}$ drachms (4 oz.); rice, 50 drachms ($5\frac{1}{2}$ oz.); and weak soup made from mutton or beef, as much as might be required.

Coffee, sugar, and tobacco were provided for the sick, if ordered by the surgeons in attendance; and the food was cooked in a manner similar to that in common use among the soldiers.

The dispensary appeared to be tolerably well furnished, but I am unacquainted with the character and quality of the drugs which were used. The surgical stores were of the most meagre description, and the instruments contained in them were insufficient for the satisfactory performance even of the minor operations.

There were two surgeons and two apothecaries resident at the hospital, and it was visited frequently by the principal surgeon of the forces.

The medical staff of the Turkish army is a very heterogenous body. Attached to it are many Germans, Hungarians, Italians, Frenchmen, etc., who have studied, or profess to have studied, in the medical schools of their respective countries. Some of these men have been driven into the Turkish service by necessity; and among them are to be found several whose talents are of no mean order, but their merits are crushed beneath the mass of ignorance with which they have to contend, and the discomforts of the degrading position in which they are generally placed; others are simply adventurers whose knowledge of medicine and qualifications to practise it are very dubious.

The Turkish medical men have been, with few exceptions, educated at Constantinople or at Alexandria. Some have a highly creditable acquaintance with their profession in its different branches; but others, and by far the greater number, are very ignorant.

The surgery which I saw practised at the Balaklava Hospital was exceedingly barbarous, and the medical practice was of a somewhat haphazard description. Preparations involving a multitude of constituents, such as were delighted in by physicians a century and a half ago, were much used; and *mithridate*, a preparation of wonderful complexity, which was considered by our forefathers to have great virtue as an antidote to poison and to pestilence, was a favourite remedy for cholera, with at least one of the hospital surgeons, a man of mature age and standing.

But it is not to be forgotten that great difficulties are experienced in teaching and acquiring scientific medicine in Turkey, from the general want among the students of that preliminary education which is essential to prepare the mind to comprehend correctly the truths of any science; from the, as yet, unmanageable character of the Turkish language as a means of conveying instruction in medicine, in consequence of the want of a scientific terminology; and from the obstacles which hinder an efficient tuition in the French language. With these impediments, the talented professors who occupy, and have occupied for some time, the different chairs at the Turkish schools of medicine, have to contend; and it is no wonder that their leaven has not yet pervaded the whole of the native members of the Army Medical Staff.

Notwithstanding the ignorance which too commonly prevails among the Turkish surgeons, there is, nevertheless, in the Turkish army, a sufficient amount of medical skill to effect considerable good among the soldiers, if it were properly fostered and supported. The medical staff seems, however, to have literally no influence in controlling those matters which most concern it. It is slighted by the Porte, and its requirements are unattended to by commanding officers. Indeed, it is probable that the short-comings of the Army Medical Staff are due as much to neglect of this kind as to the medical men themselves.

Over all that relates to the provision of food, clothing, and shelter for the sick, the medical man has little or no control; yet these things have peculiar reference to his duties, particularly during harassing campaigns, when the soldiers are most commonly broken down by diseases which arise from fatigue and want, and when the treatment required for the sick is the administration not so much of medicine as of a diet which the ordinary stores of a hospital do not usually admit of. Under such circumstances, the requisitions of the Turkish medical staff are usually unattended to.

The special requirements of the medical man are, moreover, thwarted by the character of the stores placed at his disposal. Large quantities of worthless and useless drugs and preparations, the names and uses of some of which are occasionally not known, are sent to the different depôts yearly. The surgical appurtenances, also, are exceedingly scanty and imperfect, and occasionally they are wanting almost altogether. Much, therefore, as the ignorance of the medical men has to do with the degraded and inefficient condition of the healing art in the Turkish army, it is not the sole cause of that degradation and inefficiency. The ignorance of commanding officers, and that system of official dishonesty, of which the bad condition of the medical stores is an illustration, contribute equally to these results. Any efforts for the reform of the medical staff and administration of the Turkish army, unaccompanied by reforms in the government executive will doubtless fail.

The hospital at Balaklava was under the supreme control of a military officer, a Bey, who had the power of providing whatever might be required in addition to the ordinary supplies of the hospital.

The conveyance of the sick and wounded of the Turkish army during the Crimean war was effected principally by means of Tartar wagons, buffalo wagons, and pack-animals. The Tartar wagons (few in number) were, I believe, part of the spoil of Eupatoria. They were light, four wheeled vehicles, covered in, and they answered well for sick conveyances. The buffalo wagons were vehicles of the rudest construction. They had four wheels, but the wheels rarely approximated to a circle, and each wheel generally differed from its fellow in diameter. The bottom was formed of three or four pieces of rough undressed timber, and the sides had a rude railing inclining outwards.

On the evening when the Mamelon Vert was assaulted and captured (June 9th, 1855), a division of Turks having been moved into the trenches on the right of Careening Bay ravine, an order was sent to the officer in charge of the hospital at Balaklava, directing him to send all the patients fit for removal to Eupatoria, so that every preparation could be made for the reception of any wounded men who might be sent from the front. A little before sunset, about twenty-five buffalo wagons, of the character just described, slowly ascended the road to the hospital. At the same time a movement was observed amongst the huts, and very soon about two hundred and fifty patients were, with their packs, placed on the ground in front of the huts. Many of these poor fellows were quite incapable of sitting up, unless supported. When the patients to be removed had all been got together, the process of placing them in the wagons commenced; but before the whole of the vehicles were freighted night had fallen. It was near ten o'clock when a chorus of the dismalest creaks that ungreased axles could emit, announced that the wagons had started with their loads of suffering. For several hours had the invalids, most of whom were affected with diarrhœa or fever, been kept in the open air upon the ground; then came the rough and slow journey in the wagons, and after that would be the embarkation.

The most common mode of conveying the sick was by pack-animals, the patient being placed astride of an ordinary pack-saddle. I have frequently seen invalids fall off the animals exhausted, and lie by the road-side for some time before they were again able to mount. I have witnessed instances of this kind occur within a short distance of the hospital, yet no assistance has been given; the sick man lying upon the ground, the leader of the pack-animal sitting by him, until he was able again to proceed.

About twenty mules, with seats for the sick and wounded, after the French fashion, were attached to the Turkish force before Sebastopol during a part of the war, and they did good service.

I had no means of ascertaining the average sick-rate of the Turkish army in peace, or during the Crimean war.

In the winter of 1854-55, the Turkish force at Balaklava underwent great privations, and suffered from excessive sickness.

In the summer and autumn of 1855, much sickness prevailed among the troops at Eupatoria. The hygienic condition of the town and of the entrenched camp, particularly of the former, was bad. The streets were little better than foul drains in wet weather; the court-yards were generally littered with filth, and the portions of the town which were occupied by troops, and the camp, were much crowded. The rations were deficient, fresh meat and vegetables, and the full allowance of fresh bread, being rarely issued; and the water chiefly used was brackish. Scurvy of a serious character broke out and affected many of the men, and continued fever became rife.

The Turkish force, which was encamped before Sebastopol, during the same period was plentifully rationed, and it had usually good health. In the summer, diarrhœa and cholera broke out in this force, but the diseases did not prevail to any great extent.

It is difficult to account for the good supply of food to the Turkish force before Sebastopol, while the troops at Eupatoria were so wretchedly rationed; unless it be surmised that, in the former instance, the Turkish military authorities were aided by the British Commissariat.

An interesting illustration of the capabilities of the Turkish soldier is afforded by the state of health which existed among the soldiers of the Turkish Contingent while that force was stationed at Kertch. I have been favoured with the following particulars respecting the Contingent by Dr. Milroy, one of the Commissioners appointed to inquire into the sanitary condition of her Majesty's forces in the Crimea.

The men were large and muscular, their age was generally from 20 to 25 or 28 years, and they were capable of great and continued exertion, as was shown by the large amount of labour they underwent in the formation of entrenchments; roads, etc., in addition to their ordinary field-duties. The strength of the force was somewhat more than 16,000 men, and, previously to the 1st of January, 1856, the average number of sick in hospital did not exceed 320, being under two per cent. The average number of admissions into the hospital per week was from 80 to 100, or much below one per cent. The deaths were about ten per cent. of the admissions, or at the annual rate of about 28 or 29 per 1,000 of the force. During the first five weeks of 1856 the rate of sickness increased to about 2·1 per cent., and in the second week of February scurvy appeared, and the sick rate rose to nearly five per cent.; but the mortality was not increased. The augmented rate of sickness in January and February was coincident with greater severity of the weather; and the determining causes of the scurvy were apparently an irregular supply of fresh meat and vegetables, and greater exposure. The troops which suffered most from the disease were those which were hutted in the most exposed situations. A prompt supply of fresh vegetables and lime-juice, and the removal of the affected troops to better quarters, speedily restored the health and efficiency of the force. The men were well clothed, and they received their pay regularly; their rations were similar to those furnished to the Turkish army; their habits were temperate, and, as is customary with Turkish soldiers, they drank no spirituous fluids.

"The very favourable condition of the health of the Turkish Contingent, notwithstanding some obvious sanitary defects in their camps, speaks volumes as to the preservative influence of temperance, and the disuse of intoxicating liquors, and the lesson should not be forgotten." I would add that the natural physical powers of the men, the mode in which they prepared and the times at which they eat their food, and, with the exception of their duties, the comparatively slight change between the mode of life in the camp and the habits in which they had been reared, would not be without influence in maintaining the healthy state of the force.

I had no opportunity of becoming acquainted with the barrack life of the Turkish soldier.

Bramley, near Leeds, May 1857.

